# Landforms predict phylogenetic structure on one of the world's most ancient surfaces

**DOI:** 10.1186/1471-2148-8-152

**Published:** 2008-05-19

**Authors:** Mitzy Pepper, Paul Doughty, Richard Arculus, J Scott Keogh

**Affiliations:** 1 School of Botany and Zoology, The Australian National University, Canberra, ACT 0200, Australia; 2 Department of Terrestrial Vertebrates, Western Australian Museum, 49 Kew Street, Welshpool, WA 6106, Australia; 3 School of Earth and Marine Sciences, The Australian National University, Canberra, ACT 0200, Australia

## Abstract

**Background:**

The iconic Pilbara in northwestern Australia is an ancient geological and biophysical region that is an important zone of biodiversity, endemism and refugia. It also is overlain by some of the oldest erosion surfaces on Earth, but very little is known about the patterns of biotic diversity within the Pilbara or how they relate to the landscape. We combined phylogenetic and spatial-autocorrelation genetic analyses of mitochondrial DNA data on populations of the gekkotan lizard *Lucasium stenodactylum *within the Pilbara with geological, distributional and habitat data to test the hypothesis that ancient surface geology predicts current clade-habitat associations in saxicoline animals.

**Results:**

This is the first detailed phylogenetic examination of a vertebrate organism across the Pilbara region. Our phylogeny provides strong support for a deep and ancient phylogenetic split within *L. stenodactylum *that distinguishes populations within the Pilbara region from those outside the Pilbara. Within the Pilbara region itself, our phylogeny has identified five major clades whose distribution closely matches different surface geologies of this ancient landscape. Each clade shows strong affinities with particular terrain types and topographic regions, which are directly related to different geological bedrock.

**Conclusion:**

Together our phylogenetic, distributional, geological and habitat data provide a clear example of ecological diversification across an ancient and heterogeneous landscape. Our favoured hypothesis is that ancestors of the Pilbara lineages radiated into the region at the onset of aridity in Australia approximately 5 mya and locally adapted to the various ancient and highly stable terrain types and the micro-habitats derived from them. In terms of specimen recovery and analysis, we are only beginning to reconstruct the biotic history of this ancient landscape. Our results show the geological history and the habitats derived from them will form an important part of this emerging story.

## Background

**Figure 1:**
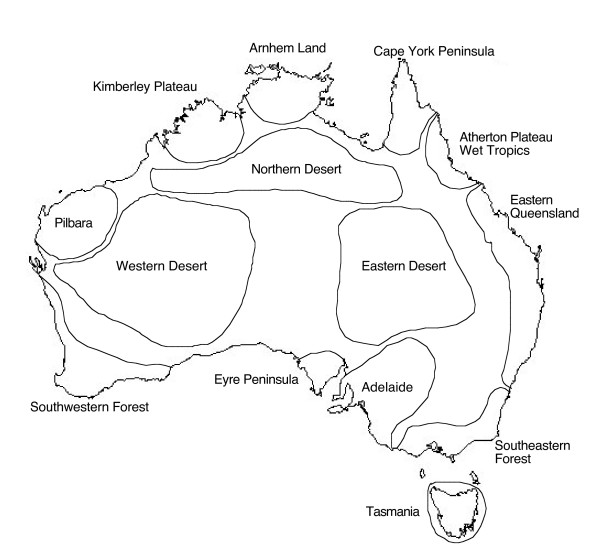
Map of Australia showing areas of animal endemism (modified from Cracraft, 1991). The iconic Pilbara region is in northwestern Australia.

The Pilbara in northwestern Australia is an ancient geological and biophysical region (Fig. 1) that has repeatedly been identified as an important zone of biological diversity, endemism and past refugia based on congruent biogeographic patterns of both fauna and flora [1-3]. Our understanding of the biotic history of the region is still in its infancy (reviewed in [4]), but the geology of the Pilbara region is well understood [5] and may serve as an important predictor of biological diversity.

**Figure 2:**
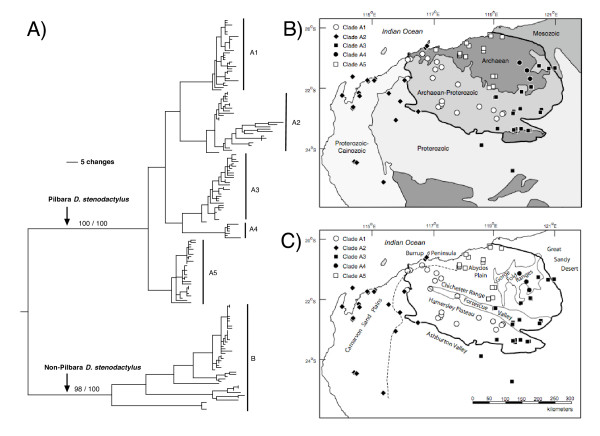
(A) Summary of phylogenetic results of the fine-scale ND2+tRNA data set of *Lucasium stenodactylum *showing the distribution of major clades in the Pilbara. Values refer to unweighted parsimony bootstraps and Bayesian posterior probabilities, respectively, of the major clades (see electronic supplementary materials for full details). (B) Simplified geological basement map of the Pilbara and surrounding regions. The geology adapted from the 1978 BMR Geological Map of Australia. The lines indicate boundaries between rock sequences of different ages, and the heaviest weighted line defines the boundary of the Pilbara Craton; (C) Outline map at same scale as (B) showing important topographic features such as sand plains, valleys, plateaus and ranges – modified from Beard (1969) and improved with space-borne altimetry. The dashed line is the 200 m contour drawn where the boundary between sand plains and elevated ground eastwards is prominent.

The Pilbara is underlain by sedimentary and igneous rocks ranging up to 3.72 billion years (Ga) in age, and overlain by some of the most ancient erosion surfaces on Earth [6]. The Pilbara Craton in the northeastern portion of the region has two parts; Archaean (3.72 to 2.85 Ga) granites and metamorphosed volcanic rocks ("greenstones") in the north, and, stratigraphically overlying these rocks in the south, is a group of basinally-deposited, younger (2.77 to 2.40 Ga) Archaean to Proterozoic iron-rich sedimentary rocks now exposed in the Hamersley Range (Fig. 2) [7]. Much younger terrains underlain by unmetamorphosed sedimentary rock types surround the Pilbara Craton. The present Pilbara land surfaces are characterized by a long history of tectonic stability and extremely slow rates of erosion [8]. Unlike the east coast of Australia where ongoing uplift commenced 80 million years ago (Ma) [9], the Pilbara has not been tectonically active since the Late Jurassic (160 Ma) when a continental sliver rifted away from the Pilbara region of northern Australia [10].

The Pilbara Craton displays remarkable topographic heterogeneity compared to the coastal plains of the southern Carnarvon and valley plains of the Ashburton regions, or the undulating dunes of the sandy deserts to the east. Major physiographic features of the Pilbara include the plateaus and ranges of the Hamersley and Chichester, and the heavily excised gorges of the Fortescue River as well as a number of other major river systems that traverse the Pilbara including the Ashburton, the De Grey and the Shaw.

Only two previous studies, both on invertebrates, have evaluated phylogenetic structure of organisms that span the Pilbara and surrounding regions [11,12]. Both have demonstrated that geological structures and complexity are fundamental predictors of phylogenetic structure for subterranean fauna, but there have been no similarly detailed studies of vertebrate animals that span the Pilbara.

*Lucasium stenodactylum *is a diplodactylid gecko with a large geographic range that includes the Pilbara region, and is part of a gekkotan radiation of Gondwanan origin [13,14]. We have detailed elsewhere the broader-level molecular systematics and biogeography of the species-group to which *L. stenodactylum *belongs, and identified an ancient split between populations distributed in the broader Pilbara region and those outside the Pilbara [4]. We are currently assessing the taxonomic status of the Pilbara populations. Here we consider *L. stenodactylum *populations within the Pilbara based on much larger and more detailed sampling, with particular reference to testing the hypothesis that ancient surface geology, and the habitats they determine, predicts phylogenetic structure in these rock dependent animals.

## Methods

Tissue samples were obtained for 169 *Lucasium stenodactylum*, of which 121 were from the Pilbara region (Table 1: See Additional file 1). These animals represent nearly all the *L. stenodactylum *specimens in tissue collections held by the Western Australian Museum (WAM), and given the current availability of specimens, exploit the maximum possible geographical sampling from the species' range. Samples from outside the Pilbara are limited in the central and northern inland parts of Australia and are considered elsewhere [4]. For all individuals a 1200-base pair (bp) region of the mitochondrial genome was sequenced that included the entire mitochondrial NADH dehydrogenase subunit 2 (ND2) gene and the flanking transfer RNA (tRNA) genes tRNA^Met ^(partial), and tRNA^Trp ^(entire), tRNA^Ala ^(entire) and tRNA^Asn ^(partial). Details of primers and lab protocols are outlined elsewhere [4]. *Lucasium squarrosum and L. wombeyi *were used as outgroups for all analyses [4]. Therefore, we used unweighted parsimony and Bayesian approaches to analyze the data. Heuristic parsimony analyses were implemented with the computer program PAUP*4.0b10. We used TBR branch swapping and ran the parsimony analysis five times from random starting points to make sure overall tree space was well searched. Bayesian analyses were run with the computer program MrBayes v3.1.2. We ran analyses on the full data set as one unit and also partitioned the data into four components: non-coding tRNA and first, second and third codons. For both sets of analyses we allowed all parameters to be estimated from the data during the runs and used the default value of four Markov chains per run. We ran the full analyses five times to make sure overall tree-space was well sampled and to avoid getting trapped in local optima. We ran each analysis for a total of 2,000,000 generations and sampled the chain every 100 generations, resulting in 40,000 sampled trees. Log-likelihood values reached a plateau after approximately 100,000 generations for both sets of analyses (1,000 sampled trees), so we discarded the first 5,000 trees as the burn-in and used the last 35,000 trees to estimate Bayesian posterior probabilities. We used the results of 10,000 "fast" unweighted non-parametric parsimony bootstrap replicates and Bayesian posterior probabilities to assess branch support.

We also used two complementary approaches to examine fine-scale genetic patterns across and within the Pilbara clades using GenAlEx 6.0 [15]. We used Mantel tests of matrix correspondence following the methods of Smouse et al. [16] to test for isolation by distance by comparing the pairwise geographic and total genetic distance matrices across all samples, and within clades, with tests of significance by 9,999 permutations. We also used a powerful micro-spatial autocorrelation technique developed by Smouse and Peakall [17] that uses pairwise geographic and genetic distance matrices to dissect out positive genetic correlation at various geographic size classes (see [18] for review; [19-21]). A significant departure from the null hypothesis of no structure (*r *= 0) is obtained when a positive *r *value falls outside of the 95% confidence interval and this is interpreted as the size of the "genetic neighbourhood" based on the data at hand. We used the combined spatial autocorrelation analysis across the five clades to provide an overall estimate of genetic neighbourhood size. We used a distance class of 10 km and evaluated 150 size classes, giving an evaluation over 150 km of space.

For all 121 individuals from the Pilbara Region, we assembled a distributional data set based on latitude and longitude for mapping with ArcGIS Desktop (ESRI). Shapefiles were created for each identified Pilbara clade from our phylogeny and viewed in ArcMap. Distributions were then overlain on to the Geological Regions Dataset obtained from Geoscience Australia [22]. This dataset was derived from the 1978 BMR 1:2,500,000 Geological Map of Australia and includes metadata such as the age, landform and stratigraphy of different geographical regions within the Australian continent. The distributions of the major Pilbara clades also were examined with respect to the land types on which they occur, based on a recent inventory and condition survey of the Pilbara that takes into account landforms, soils, vegetation and drainage patterns [23]. These terrain types relate fundamentally to the basement geology.

## Results and Discussion

After the exclusion of unalignable regions in the tRNAs, the edited alignment comprised 1142 characters and of these 610 (53%) were variable and parsimony informative. We used ModelTest [24] to compare model parameter estimates with those generated for the Bayesian analyses in Mr. Bayes. Both the hierarchical likelihood ratio tests and the Akaike information criterion in ModelTest supported TrN+I+G model as the best-fit substitution model for the combined data set and gave a -lnL = 7334.9434. The estimated parameters were as follows: nucleotide frequencies A = 0.3422, C = 0.3387, G = 0.0734, T = 0.2457; substitution rates A↔C 1.000, A↔G 32.4198, A↔T 1.000, C↔G 1.000, C↔T 14.7978, G↔T 1.0000; proportion of invariant sites = 0.4750; gamma shape parameter = 0.1.1596. The Bayesian analyses produced parameter estimates that were very similar to those produced by ModelTest (not shown).

Unweighted parsimony analyses and both unpartitioned and partitioned Bayesian analyses produced very similar topologies. Figure 2a shows one of 100 most parsimonious trees saved during the unweighted parsimony analysis. Parsimony bootstrap support values and unpartitioned Bayesian posterior probabilities are shown for major branches only, but the results of our parsimony bootstrap and unpartitioned Bayesian analyses with details of each individual sample are shown in Additional File 2*PepperEtAlBootstrap *and Additional File 3*PepperEtAlBayesian *(results for the partitioned Bayesian analyses were virtually identical to the unpartitioned and are not shown).

The ND2+tRNA data set shows strong support for the deep and old phylogenetic split between populations in the Pilbara region (Clade A, bootstrap value 100, posterior probability 100) and non-Pilbara populations (Clade B, bootstrap value 100, posterior probability 100), in agreement with what we found based on a reduced number of samples, but additional genes [4]. This result is consistent with the stark geological contrast between the ancient and exposed surface landscape and topography in the Pilbara Craton relative to the surrounding dunefields of the desert regions. For example, the Archaean to Proterozoic igneous (granites and greenstones) and sedimentary rocks (ironstones and cherts) form rugged outcrops at the surface in the Pilbara, and are elevated compared with surrounding regions. The lower-lying area to the south and east of the Pilbara Craton is generally overlain by much younger sedimentary rocks of Proterozoic to Cainozoic age that have given rise to sandplains and dunefields [25].

The ND2+tRNA data set also identified five major clades within the Pilbara endemic *L. stenodactylum *Clade A, with generally high bootstrap values and posterior probabilities (Clade A1 bootstrap value = 98, posterior probability = 100; Clade A2 bootstrap value = 94, posterior probability = 100; Clade A3 bootstrap value = 90, posterior probability = 100; Clade A4 bootstrap value = 100, posterior probability = 100; Clade A5 has a high bootstrap value of 93 but the clade was not monophyletic in the Bayesian analysis). Uncorrected genetic divergence between clades ranged from 3.89% between A3 and A4 to 5.80% between A2 and A5, and maximum uncorrected divergence within each clade were as follows, A1: 1.49%, A2: 3.16%, A3: 1.75%, A4: 1.12%, A5: 1.67%. Our genetic data suggests that the present-day lineages within the Pilbara are relatively young with inter-clade divergences dating to approximately 3 – 4.5 mya and maximum intra-clade divergences dating to 0.8 – 2.4 mya using a standard but simplistic reptile 1.3%/my mtDNA clock [26]. Non-Pilbara Clade B exhibits deep genetic structure but this is not the focus of this paper (see [4]).

Our phylogeny demonstrates that clade distributions are remarkably correlated with geological (Figure 2b) and topographic (Figure 2c) domains at both a large geographic scale (craton versus non-craton) and also at the level of fine intra-craton provinciality. Clades A1, A3, A4 and A5 are predominantly restricted to the rugged Pilbara Craton whereas Clade A2 is mostly confined to the lower-lying non-craton, for example the Carnarvon Sand Plains west of the Craton. However, it is notable while Clade A2 extends eastwards of the 200 m contour from the Sand Plains, this Clade does not extend onto the Craton at an identical elevation (Figure 2c); the additional distributional controlling factor may be the contrast in exposed rock types and their associated habitats. Within the Pilbara Craton, Clade A1 is restricted to the Hamersley Plateau and other upland regions in the Archaean-Proterozoic ironstone units in the south of the Craton. Clade A5 is restricted to the sandy Abydos plain developed upon the Archean granite unit in the north of the Craton and is bound to the east by the distinctive Gorge Fold Ranges, a series of parallel, razor-back fold ridges, rising up to 80 m above the surrounding terrain [27] (Figure 2c). Clade A4 also is found in the far easternmost part of this granite unit, but appears to be associated with the ridges of the Gorge Fold Ranges. Although Clade A3 is distributed throughout both the Archaean-Proterozoic ironstone and the Archean granite unit in the eastern portion of the Craton, this clade is absent from the ranges and occupies the lowlands in the eastern part of the Craton, extending its distribution up the Fortescue Valley. The only clade found outside the Pilbara Craton is Clade A2, which is almost exclusively associated with much younger sandstone units that dominate the low-lying Carnarvon Sand Plains along the northwestern Australian coastline. A small number of individuals from this clade are found within the Pilbara Craton on the Burrup Peninsula, but these individuals are geographically connected to the rest of the clade by the low-lying sandplains that stretch along the coast. A number of major rivers traverse the Pilbara, but the distribution of clades suggests these generally dry riverbeds are not current barriers to dispersal in these animals.

**Figure 3:**
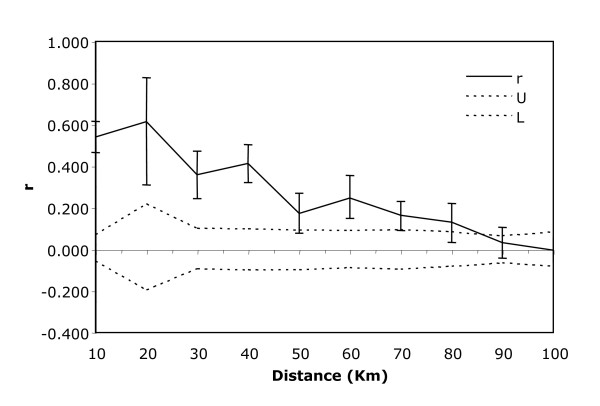
Correlogram showing the results of the fine-scale spatial autocorrelation analysis where genetic correlation values (*r*) are plotted as a function of distance. U and L represent 95% confidence intervals around the null hypotheses of no structure. The plot shows that there is a significant positive genetic structure at all distances classes less than 90 km distance class.

Each clade exhibits a strong preference for a particular terrain type, each of which are related directly to underlying geological structure. For example, Clades A1 and A3 are associated with stony lower slopes and plains, while Clade A2 is associated with sandplains, dunes and clay pans. Clade A4 also prefers stony lower slopes and plains while Clade A5 is distributed between sandplains, dunes and claypans and stony lower slopes and plains. A Mantel test across all five clades showed evidence of isolation by distance in the genetic data (Mantel, *R*_xy _= 0.470, P = 0.010). Strong isolation by distance also was detected in four of the five individual clades (A1: *R*_xy _= 0.141, P = 0.030; A2: *R*_xy _= 0.702, P = 0.010; A3: *R*_xy _= 0.468, P = 0.010; A5: *R*_xy _= 0.625, P = 0.010), the exception being clade A4, which has too few samples to do a meaningful test. The combined spatial autocorrelation analysis showed a significantly positive *r *value at all distance classes less than 90 km (Figure 3), which demonstrates that the spatial genetic structure varies on a regional scale that matches the size and distribution of available habitats.

## Conclusion

Together our phylogenetic, distributional, geological and habitat data provide a textbook example of ecological diversification across an ancient and heterogeneous landscape. Basement geology and superimposed topography have determined the varied micro-habitats available across the Pilbara. Each of the different types of habitats occupied by these lizards will experience quite different temperature and humidity regimes, each of which should contribute strongly to distribution of clades. While there are a variety of possible explanations, our favoured hypothesis is that ancestors of the Pilbara lineages radiated into the region at the onset of aridity in Australia approximately 5 mya and locally adapted to these ancient and highly stable terrain types and the micro-habitats derived from them. Our results are broadly consistent with patterns revealed by recent detailed phylogenetic work on subterranean diving beetles [11] and amphipods [12] in the Pilbara region. The Pilbara is well known as important an important area for refugia with high levels of endemism in a variety of plants and animals [1-3], but we are only just now beginning to reconstruct the biotic history of this ancient and complex landscape. The geological history and the habitats and micro-environments derived from geological formations will form an important part of this emerging story.

## Authors' contributions

MP carried out all the molecular genetic work and assembled and analysed the distributional data. PD provided access to tissue samples and participated in the design of the project. RA provided expertise on the geology of the Pilbara region. JSK carried out the phylogenetic and spatial genetic analyses and participated in the design of the project. MP and JSK wrote the manuscript. All authors have read and approved the manuscript.

## Supplementary Material

Additional file 1 Table 1 Click here for file

Additional file 2 PepperEtAlBootstrap Click here for file

Additional file 3 PepperEtAlBayesian Click here for file
