# Supplementary material for: Landforms predict phylogenetic structure on one of the world's most ancient surfaces
Source: BMC Evol Biol. 2008 May 19;8:152. doi: 10.1186/1471-2148-8-152 (PMC2397392; doi:10.1186/1471-2148-8-152)
Supplement: Additional file 1 — Table 1 [file 1471-2148-8-152-S1.doc]

**Table 1.** Locality information for all individuals sampled in this study.

| **Taxon** | **Lab ID#** | **Museum** | **Museum #** | **Latitude (S)** | **Longitude (E)** | **Locality (nearest)** |
| --- | --- | --- | --- | --- | --- | --- |
| *Lucasium squarrosum* | Gko 091 | WAM | 112109 | 29° 17' 09" | 117° 28' 51" | Paynes Find, WA |
| *Lucasium wombeyi* | Gko 221 | WAM | 114916 | 23° 15' 00" | 119° 53' 00" | Newman, WA |
| *Lucasium stenodactylum* | Gko 101 | WAM | 90635 | 21° 36' 34" | 118° 58' 28" | Woodstock Station, WA |
|  | Gko 102 | WAM | 90671 | 21° 36' 34" | 118° 58' 28" | Woodstock Station, WA |
|  | Gko 103 | WAM | 90702 | 21° 36' 35" | 119° 01' 17" | Woodstock Station, WA |
|  | Gko 104 | WAM | 97256 | 23° 23' 00" | 120° 09' 00" | Wheelarra Hill, WA |
|  | Gko 105 | WAM | 97257 | 23° 23' 00" | 120° 09' 00" | Wheelarra Hill, WA |
|  | Gko 106 | WAM | 102053 | 20° 19' 00" | 119° 15' 00" | Degrey River, WA |
|  | Gko 107 | WAM | 102181 | 22° 36' 00" | 118° 30' 43" | Mount Windell, WA |
|  | Gko 108 | WAM | 102185 | 22° 36' 00" | 118° 30' 43" | Mount Windell, WA |
|  | Gko 109 | WAM | 102481 | 23° 02' 41" | 115° 47' 14" | Barlee Range Nature Reserve, WA |
|  | Gko 111 | WAM | 102752 | 24° 31' 55" | 120° 17' 28" | Little Sandy Desert, WA |
|  | Gko 112 | WAM | 104001 | 21° 36' 42" | 118° 57' 20" | Woodstock Station, WA |
|  | Gko 113 | WAM | 106155 | 23° 52' 00" | 118° 34' 00" | Turee Creek Homestead, WA |
|  | Gko 114 | WAM | 108799 | 18° 56' 26" | 128° 47' 04" | Banana Springs, WA |
|  | Gko 115 | WAM | 108800 | 17° 15' 00" | 128° 18' 00" | Bream Gorge, WA |
|  | Gko 116 | WAM | 108803 | 19° 42' 00" | 121° 14' 00" | East of Sandfire Roadhouse, WA |
|  | Gko 117 | WAM | 108807 | 18° 09' 00" | 122° 33' 00" | Thangoo Homestead, WA |
|  | Gko 118 | WAM | 108835 | 24° 42' 00" | 119° 36' 00" | Kumarina, WA |
|  | Gko 119 | WAM | 108846 | 21° 02' 00" | 116° 49' 00" | Cherralta Homestead, WA |
|  | Gko 120 | WAM | 108847 | 21° 02' 00" | 116° 49' 00" | Cherralta Homestead, WA |
|  | Gko 121 | WAM | 110584 | 19° 53' 21" | 128° 51' 36" | Tanami Desert, WA |
|  | Gko 122 | WAM | 110588 | 19° 51' 25" | 128° 50' 53" | Tanami Desert, WA |
|  | Gko 124 | WAM | 110591 | 19° 53' 55" | 128° 51' 57" | Tanami Desert, WA |
|  | Gko 125 | WAM | 110592 | 19° 35' 36" | 128° 51' 40" | Tanami Desert, WA |
|  | Gko 126 | WAM | 110593 | 19° 53' 59" | 128° 49' 37" | Tanami Desert, WA |
|  | Gko 127 | WAM | 110706 | 22° 35' 19" | 117° 14' 11" | Mount Brockman, WA |
|  | Gko 128 | WAM | 110710 | 22° 37' 03" | 117° 10' 53" | Mount Brockman, WA |
|  | Gko 129 | WAM | 110723 | 22° 26' 16" | 117° 17' 04" | Mount Brockman, WA |
|  | Gko 130 | WAM | 110741 | 22° 31' 05" | 117° 17' 04" | Mount Brockman, WA |
|  | Gko 131 | WAM | 112692 | 21° 41' 16" | 115° 05' 31" | Onslow, WA |
|  | Gko 132 | WAM | 112964 | 17° 03' 35" | 122° 43' 00" | Broome, WA |
|  | Gko 133 | WAM | 112974 | 17° 05' 01" | 122° 39' 35" | Broome, WA |
|  | Gko 134 | WAM | 113026 | 20° 16' 50" | 118° 52' 50" | Lesley Salt Works, WA |
|  | Gko 135 | WAM | 113031 | 20° 14' 50" | 118° 50' 50" | Lesley Salt Works, WA |
|  | Gko 136 | WAM | 113181 | 24° 42' 00" | 119° 35' 00" | Kumarina, WA |
|  | Gko 137 | WAM | 113194 | 23° 20' 00" | 119° 52' 00" | Newman, WA |
|  | Gko 138 | WAM | 113229 | 21° 42' 00" | 116° 10' 00" | Deepdale, WA |
|  | Gko 140 | WAM | 113591 | 21° 59' 00" | 118° 50' 00" | Auski Roadhouse, WA |
|  | Gko 141 | WAM | 113601 | 21° 59' 00" | 118° 50' 00" | Auski Roadhouse, WA |
|  | Gko 142 | WAM | 114210 | 16° 24' 00" | 122° 56' 00" | Cape Leveque, WA |
|  | Gko 143 | WAM | 114211 | 16° 24' 00" | 122° 56' 00" | Cape Leveque, WA |
|  | Gko 144 | WAM | 114234 | 17° 22' 00" | 122° 09' 00" | Coulomb Nature Reserve, WA |
|  | Gko 145 | WAM | 114918 | 21° 10' 00" | 119° 49' 00" | Marble Bar, WA |
|  | Gko 146 | WAM | 114919 | 21° 10' 00" | 119° 49' 00" | Marble Bar, WA |
|  | Gko 147 | WAM | 114920 | 21° 10' 00" | 119° 49' 00" | Marble Bar, WA |
|  | Gko 148 | WAM | 115245 | 21° 38' 00" | 114° 23' 00" | South Muiron Island, WA |
|  | Gko 149 | WAM | 119277 | 26° 26' 00" | 117° 09' 00" | Mount Levra Station, WA |
|  | Gko 152 | WAM | 122758 | 24° 24' 20" | 114° 26' 39" | Manberry, WA |
|  | Gko 153 | WAM | 123831 | 25° 05' 17" | 115° 22' 48" | Gascoyne Junction, WA |
|  | Gko 154 | WAM | 125017 | 22° 42' 35" | 118° 58' 55" | Yandicoogina, WA |
|  | Gko 155 | WAM | 125031 | 22° 42' 35" | 118° 58' 55" | Yandicoogina, WA |
|  | Gko 156 | WAM | 125037 | 22° 43' 14" | 118° 59' 26" | Yandicoogina, WA |
|  | Gko 158 | WAM | 126773 | 24° 26' 35" | 114° 30' 42" | Lake McLeod, WA |
|  | Gko 159 | WAM | 127444 | 21° 45' 00" | 115° 05' 00" | Onslow, WA |
|  | Gko 160 | WAM | 127493 | 21° 44' 00" | 115° 05' 00" | Onslow, WA |
|  | Gko 161 | WAM | 127515 | 21° 44' 00" | 115° 05' 00" | Onslow, WA |
|  | Gko 162 | WAM | 127518 | 21° 44' 00" | 115° 05' 00" | Onslow, WA |
|  | Gko 163 | WAM | 127703 | 22° 47' 57" | 117° 46' 35" | Mount Tom Price Mine, WA |
|  | Gko 164 | WAM | 127776 | 22 °47' 57" | 117° 46' 35" | Mount Tom Price Mine, WA |
|  | Gko 165 | WAM | 129656 | 22° 59' 45" | 119° 05' 30" | Newman, WA |
|  | Gko 166 | WAM | 131012 | 21° 08' 18" | 117° 06' 57" | Millstream-Chitchester National Park, WA |
|  | Gko 167 | WAM | 131487 | 23° 22' 18" | 119° 37' 44" | Mount Whaleback, WA |
|  | Gko 168 | WAM | 132239 | 21° 46' 40" | 114° 52' 14" | Urala Station, WA |
|  | Gko 169 | WAM | 132240 | 21° 45' 42" | 114° 51' 10" | Urala Station, WA |
|  | Gko 170 | WAM | 132528 | 20° 40' 20" | 116° 45' 22" | Burrup Peninsula, WA |
|  | Gko 171 | WAM | 132533 | 20° 40' 20" | 116° 45' 22" | Burrup Peninsula, WA |
|  | Gko 172 | WAM | 132556 | 20° 37' 34" | 116° 47' 23" | Burrup Peninsula, WA |
|  | Gko 173 | WAM | 132726 |  |  |  |
|  | Gko 174 | WAM | 132759 | 16° 01' 21" | 128° 52' 07" | Lake Argyle, WA |
|  | Gko 175 | WAM | 135006 | 23° 20' 15" | 119° 41' 48" | Mount Whaleback, WA |
|  | Gko 176 | WAM | 135007 | 23° 24' 11" | 119° 41' 46" | Mount Whaleback, WA |
|  | Gko 177 | WAM | 135319 | 20° 45' 16" | 117° 04' 52" | Cape Lambert, WA |
|  | Gko 178 | WAM | 139043 | 19° 48' 44" | 121° 28' 25" | Mandora, WA |
|  | Gko 179 | WAM | 139232 | 21° 15' 59" | 120° 27' 19" | Meentheena Homestead, WA |
|  | Gko 180 | WAM | 140755 | 15° 43' 00" | 128° 44' 00" | Kununurra, WA |
|  | Gko 182 | WAM | 145077 | 27° 03' 07" | 125° 09' 31" | Officer Basin, WA |
|  | Gko 183 | WAM | 145195 | 22° 14' 35" | 114° 02' 05" | Learmonth Airstrip, WA |
|  | Gko 184 | WAM | 145196 | 22° 14' 35" | 114° 02' 05" | Learmonth Airstrip, WA |
|  | Gko 185 | WAM | 145449 | 26° 15' 45" | 121° 30' 39" | Lorna Glen Station, WA |
|  | Gko 186 | WAM | 145566 | 20° 42' 00" | 118° 38' 24" | Port Headland, WA |
|  | Gko 187 | WAM | 145592 | 20° 46' 48" | 118° 38' 24" | Port Headland, WA |
|  | Gko 188 | WAM | 145703 | 22° 49' 15" | 119° 17' 13" | Weeli Wolli Creek, WA |
|  | Gko 189 | WAM | 145704 | 22° 50' 02" | 119° 16' 22" | Weeli Wolli Creek, WA |
|  | Gko 190 | WAM | 145742a | 21° 56' 24" | 118° 57' 39" | Chitchester Range, WA |

|  | Gko 191 | WAM | 145742b | 21° 56' 24" | 118° 57' 39" | Chitchester Range, WA |
| --- | --- | --- | --- | --- | --- | --- |
|  | Gko 192 | WAM | 151021 | 15° 56' 00" | 128° 54' 00" | Kununurra, WA |
|  | Gko 195 | WAM | 151440a | 26° 12' 03" | 121° 18' 13" | Lorna Glen Station, WA |
|  | Gko 196 | WAM | 151440b | 26° 12' 03" | 121° 18' 13" | Lorna Glen Station, WA |
|  | Gko 197 | WAM | 151484a | 26° 15' 45" | 121° 30' 39" | Lorna Glen Station, WA |
|  | Gko 198 | WAM | 151484b | 26° 15' 45" | 121° 30' 39" | Lorna Glen Station, WA |
|  | Gko 199 | WAM | 151743 | 21° 56' 24" | 118° 57' 39" | Chitchester Range, WA |
|  | Gko 200 | WAM | 154249 | 22° 17' 57" | 119° 01' 22" | Munjina Roadhouse, WA |
|  | Gko 201 | WAM | 154560 | 23° 22' 45" | 120° 06' 19" | Newman, WA |
|  | Gko 202 | WAM | 157145 | 22° 42' 21" | 120° 32' 17" | Roy Hill, WA |
|  | Gko 203 | WAM | 157156 | 22° 47' 02" | 120° 30' 02" | Roy Hill, WA |
|  | Gko 204 | WAM | 157189 | 22° 09' 51" | 114° 35' 23" | Yanrey, WA |
|  | Gko 205 | WAM | 157273 | 22° 15' 27" | 114° 36' 00" | Yanrey, WA |
|  | Gko 206 | WAM | 157539 | 21° 44' 52" | 116° 04' 31" | Pannawonica, WA |
|  | Gko 207 | WAM | 157573 | 21° 44' 52" | 116° 04' 31" | Pannawonica, WA |
|  | Gko 208 | WAM | 157732 | 19° 34' 46" | 128° 52' 05" | Larranganni Bluff, WA |
|  | Gko 209 | WAM | 157733 | 19° 34' 46" | 128° 52' 05" | Larranganni Bluff, WA |
|  | Gko 210 | WAM | 157945 | 23° 17' 54" | 122° 42' 06" | Lake Views, WA |
|  | Gko 211 | WAM | 157946 | 23° 17' 54" | 122° 42' 06" | Lake Views, WA |
|  | Gko 212 | WAM | 157947 | 23° 14' 03" | 122° 42' 05" | Lake Disappointment, WA |
|  | Gko 213 | WAM | 157948 | 23° 14' 03" | 122° 42' 05" | Lake Disappointment, WA |
|  | Gko 214 | WAM | 157949 | 23° 21' 07" | 122° 40' 00" | Savory Creek Mouth, WA |
|  | Gko 215 | WAM | 157950 | 23° 21' 07" | 122° 40' 00" | Savory Creek Mouth, WA |
|  | Gko 216 | WAM | 158319 | 22° 39' 28" | 114° 23' 31" | Giralia, WA |
|  | Gko 217 | WAM | 158320 | 22° 37' 18" | 114° 23' 33" | Giralia, WA |
|  | Gko 300 | AM | R140585 | 17° 58' 36" | 122° 20' 37" | Roebuck Bay, Broome Bird Observatory, WA |
|  | Gko 301 | AM | R139899 | 16° 01' 07" | 128° 00' 14" | El Questro Station, WA |
|  | Gko 302 | AM | R155220 | 29° 02' 40" | 141° 18' 26" | Sturt NP, NSW |
|  | Gko 303 | AM | R155229 | 29° 08' 59" | 141° 07' 06" | Sturt NP, NSW |
|  | Gko 304 | AM | R155230 | 29° 08' 59" | 141° 07' 06" | Sturt NP, NSW |
|  | Gko 305 | AM | R155245 | 29° 02' 40" | 141° 18' 26" | Sturt NP, NSW |
|  | Gko 306 | AM | R155372 | 29° 02' 40" | 141° 18' 26" | Sturt NP, NSW |
|  | Gko 307 | AM | R139897 | 16° 01' 07" | 128° 00' 14" | El Questro Station, WA |
|  | Gko 313 | WAM | R110130 | 21° 52' 56.28" | 117° 47' 39.7" | PE02*, WA |
|  | Gko 314 | WAM | R110195 | 21° 23' 02.70" | 117° 03' 38.8" | PW12*, WA |
|  | Gko 315 | WAM | R110251 | 22° 40' 30.70" | 119° 50' 26.8" | RHNE05*, WA |
|  | Gko 316 | WAM | R110270 | 22° 07' 04.40" | 119° 52' 35.3" | RHNE0*, WA |
|  | Gko 317 | WAM | R110294 | 22° 40' 30.70" | 119° 50' 26.8" | RHNE05*, WA |
|  | Gko 318 | WAM | R110298 | 22° 45' 57.90" | 119° 37' 50.5" | RHNE02*,WA |
|  | Gko 319 | WAM | R110305 | 22° 49' 11.40" | 119° 36' 50.0" | RHNE01*, WA |
|  | Gko 320 | WAM | R110310 | 22° 40' 30.70" | 119° 50' 26.8" | RHNE05*, WA |
|  | Gko 321 | WAM | R158064 | 21° 18' 36.36" | 117° 16' 32.2" | PW13*, WA |
|  | Gko 322 | WAM | R158067 | 21° 40' 39.06" | 116° 58' 29.9" | PW06*, WA |
|  | Gko 323 | WAM | R158092 | 21° 23' 02.70" | 117° 03' 38.8" | PW12*, WA |
|  | Gko 324 | WAM | R158093 | 21° 23' 02.70" | 117° 03' 38.8" | PW12*, WA |
|  | Gko 325 | WAM | R158186 | 22° 17' 40.30" | 119° 03' 40.9" | RHNW11*, WA |
|  | Gko 327 | WAM | R159841 | 20° 56' 59.64" | 117° 51' 00.6" | DRE11*, WA |
|  | Gko 328 | WAM | R159850 | 20° 36' 26.28" | 118° 09' 25.2" | DRE07*, WA |
|  | Gko 329 | WAM | R159851 | 20° 31' 31.86" | 118° 04' 38.2" | DRE06*, WA |
|  | Gko 331 | WAM | R159884 | 20° 55' 10.86" | 117° 51' 39.3" | DRE10*, WA |
|  | Gko 332 | WAM | R159891 | 20° 52' 11.58" | 117° 51' 30.5" | DRE09*, WA |
|  | Gko 333 | WAM | R159895 | 20° 56' 59.64" | 117° 51' 00.6" | DRE11*, WA |
|  | Gko 334 | WAM | R159906 | 20° 58' 09.90" | 118° 02' 52.5" | DRE13*, WA |
|  | Gko 338 | WAM | R159919 | 20° 52' 11.58" | 117° 51' 30.5" | DRE09*, WA |
|  | Gko 339 | WAM | R159920 | 20° 52' 11.58" | 117° 51' 30.5" | DRE09*, WA |
|  | Gko 342 | WAM | R159941 | 20° 55' 10.86" | 117° 51' 39.3" | DRE10*, WA |
|  | Gko 343 | WAM | R159942 | 20° 55' 10.86" | 117° 51' 39.3" | DRE10*, WA |
|  | Gko 346 | WAM | R160024 | 20° 31' 31.86" | 118° 04' 38.2" | DRE06*, WA |
|  | Gko 347 | WAM | R160061 | 21° 17' 18.40" | 121° 14' 15.4" | NE12*, WA |
|  | Gko 348 | WAM | R160062 | 21° 17' 41.40" | 121° 15' 50.2" | NE13*, WA |
|  | Gko 349 | WAM | R160086 | 21° 19' 32.10" | 120° 58' 12.8" | NE08*, WA |
|  | Gko 350 | WAM | R160092 | 21° 17' 41.40" | 121° 15' 50.2" | NE13*, WA |
|  | Gko 351 | WAM | R160123 | 21° 19' 32.10" | 120° 58' 12.8" | NE08*, WA |
|  | Gko 352 | WAM | R160127 | 21° 17' 41.40" | 121° 15' 50.2" | NE13*, WA |
|  | Gko 353 | WAM | R160147 | 21° 19' 32.10" | 120° 58' 12.8" | NE08*, WA |
|  | Gko 355 | WAM | R160151 | 21° 20' 13.60" | 120° 46' 10.2" | NE05*, WA |
|  | Gko 356 | WAM | R160159 | 21° 58' 46.70" | 120° 13' 25.2" | NW01*, WA |
|  | Gko 357 | WAM | R160160 | 21° 58' 46.70" | 120° 13' 25.2" | NW01*, WA |
|  | Gko 358 | WAM | R160161 | 21° 23' 33.40" | 120° 04' 20.2" | NW12*, WA |
|  | Gko 359 | WAM | R160183 | 21° 56' 53.00" | 120° 11' 37.2" | NW02*, WA |
|  | Gko 360 | WAM | R160204 | 21° 40' 39.90" | 120° 09' 18.7" | NW05*, WA |
|  | Gko 361 | WAM | R160212 | 21° 23' 33.40" | 120° 04' 20.2" | NW12*, WA |
|  | Gko 362 | WAM | R160227 | 21° 40' 39.90" | 120° 09' 18.7" | NW05*, WA |
|  | Gko 363 | WAM | R160231 | 21° 24' 25.80" | 120° 04' 16.8" | NW11*, WA |
|  | Gko 366 | WAM | R160930 | 20° 52' 28.44" | 116° 38' 55.1" | DRW04*, WA |
|  | Gko 367 | WAM | R160931 | 21° 03' 46.40" | 116° 13' 59.9" | DRW08*, WA |
|  | Gko 369 | WAM | R160979 | 20° 52' 28.44" | 116° 38' 55.1" | DRW04*, WA |
|  | Gko 370 | WAM | R161022 | 21° 03' 18.70" | 116° 15' 05.2" | DRW07*, WA |
|  | Gko 371 | WAM | R161050 | 20° 52' 28.44" | 116° 38' 55.1" | DRW04*, WA |
|  | Gko 373 | WAM | R161064 | 21° 03' 18.70" | 116° 15' 05.2" | DRW07*, WA |
|  | Gko 375 | WAM | R161075 | 21° 04' 14.80" | 116° 12' 22.5" | DRW09*, WA |
|  | Gko 376 | WAM | R161152 | 22° 40' 30.70" | 119° 50' 26.8" | RHNE05*, WA |
|  | Gko 378 | WAM | R161992 | 22° 10' 13.10" | 115° 33' 33.9" | WYW03*, WA |
|  | Gko 381 | WAM | R162055 | 22° 26' 00.70" | 115° 55' 48.0" | WYE06*, WA |
|  | Gko 382 | WAM | R162123 | 22° 46' 23.00" | 116° 31' 19.3" | WYE02*, WA |
|  | Gko 383 | WAM | R162205 | 23° 19' 09.70" | 119° 06' 05.1" | RHNC02*, WA |
|  | Gko 386 | WAM | R162257 | 22° 50' 16.00" | 119° 16' 19.3" | RHNC13*, WA |

Lab identification numbers (Lab ID#) were given to each sample and used in the figures. Museum # refers to the voucher specimens held in the Australian Museum (AM) and the Western Australian Museum (WAM). Locality information is given to the nearest named location as provided by the museums. Localities marked with “*” refer to sample sites from the Pilbara Biological Surveys (2004) & 2 (2005) (unpublished reports). All samples listed were sequenced for the ND2+tRNA region while samples marked with “**” refer to those individuals where sequence data also was obtained for 16s and RAG-1 (Pepper et al., 2006).
