# Supplementary material for: Landforms predict phylogenetic structure on one of the world's most ancient surfaces
Source: BMC Evol Biol. 2008 May 19;8:152. doi: 10.1186/1471-2148-8-152 (PMC2397392; doi:10.1186/1471-2148-8-152)

Additional File 1. Bootstrap consensus tree based on 10,000 unweighted parsimony bootstrap replicates.

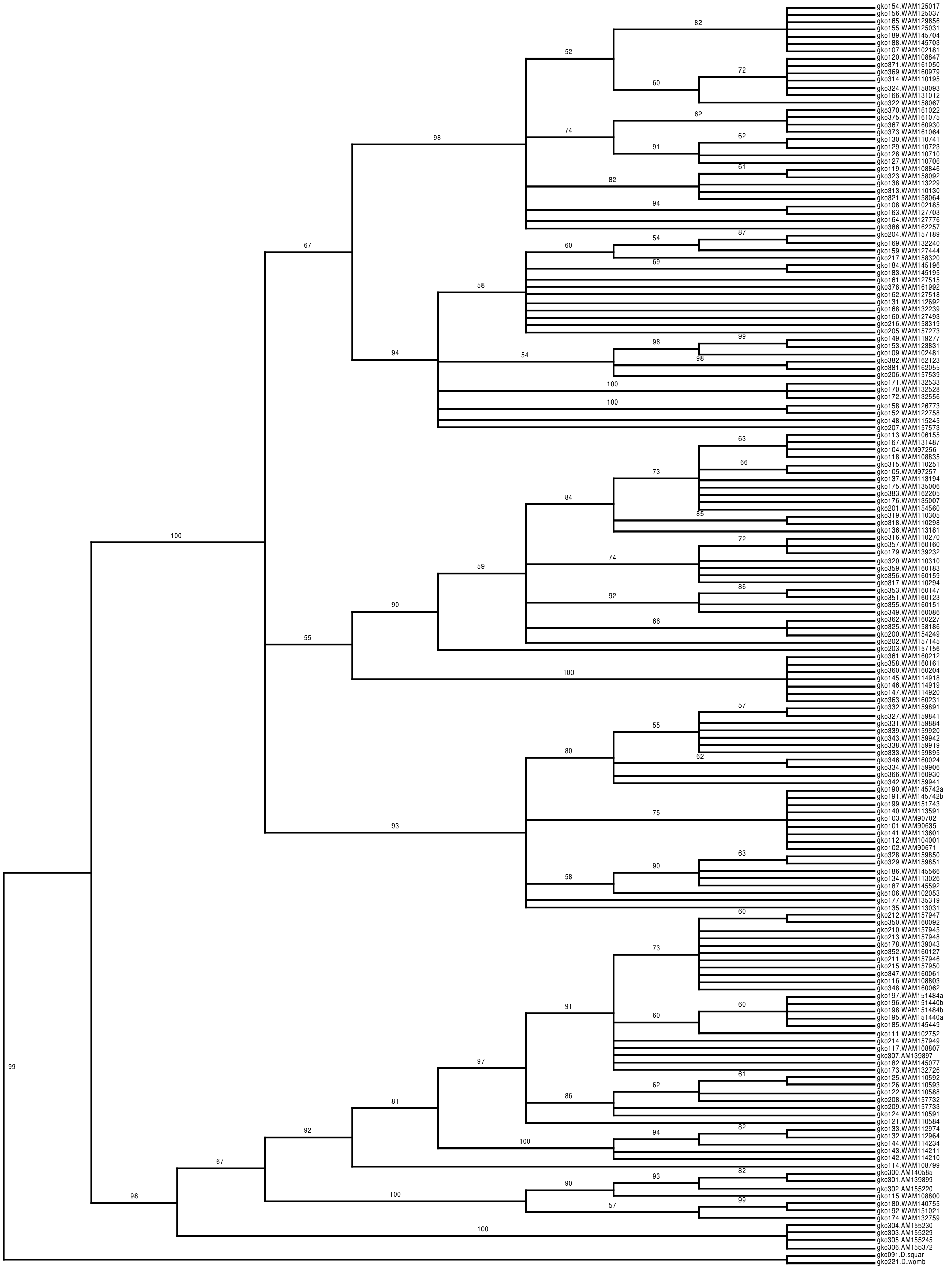

Supplement: Additional file 2 — PepperEtAlBootstrap [file 1471-2148-8-152-S2.pdf]
