# Supplementary figures and images for: Landforms predict phylogenetic structure on one of the world's most ancient surfaces
Source: BMC Evol Biol. 2008 May 19;8:152. doi: 10.1186/1471-2148-8-152 (PMC2397392; doi:10.1186/1471-2148-8-152)

2  
3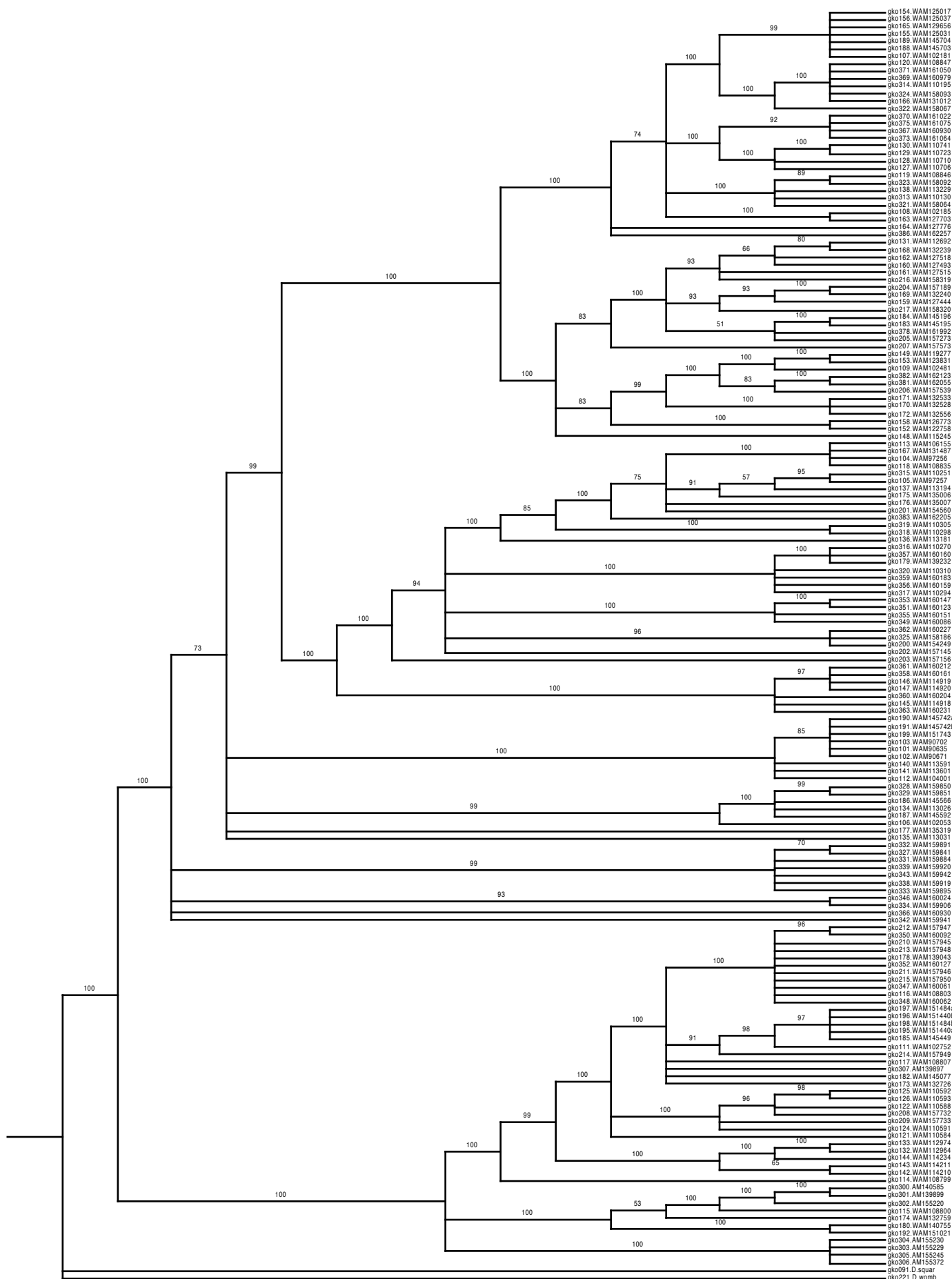

Supplement: Additional file 3 — PepperEtAlBayesian [file 1471-2148-8-152-S3.pdf]
